# Catatonia and dementia: a case report

**DOI:** 10.1192/j.eurpsy.2022.1674

**Published:** 2022-09-01

**Authors:** E. Almeida, J. Abreu, J. Martins, R. Vaz, R. Sousa, J. Brás, A. Costa, D. Teixeira, A. Marques, E. Monteiro

**Affiliations:** Centro Hospitalar Tondela-Viseu , Departamento De Psiquiatria E Saúde Mental, Viseu, Portugal

**Keywords:** Catatonia, demencia

## Abstract

**Introduction:**

Catatonia is a neuropsychiatric disorder characterized by motor, behavioral and autonomic changes. It is associated with several psychiatric disorders, including dementia. Catatonia is an underdiagnosed syndrome, so it is important to draw attention to it. Here, we review a case of a patient admitted to our psychiatric department with a clinical presentation compatible with catatonia. After proper treatment, further assessment revealed dementia.

**Objectives:**

This work aims to describe a case of catatonia in a patient with dementia.

**Methods:**

Bibliographic research using Pubmed®. Clinical file consultation and patient interviews.

**Results:**

Catatonia is a disorder that was already been described as part of several types of dementia. We present a 69-year-old female patient, admitted to our psychiatric department with clinical presentation compatible with catatonia. To admission, she presented some typical complications resulting from long immobility such as pressure ulcers and nutritional deficiencies. During the hospitalization, she developed a urinary infection and there was the need to tube feeding. She was treated with benzodiazepines and improved. Further assessment revealed dementia.

**Conclusions:**

Catatonia in dementia is not uncommon, although it is an underdiagnosed syndrome, and when treated early and properly it has a good prognosis.

**Disclosure:**

No significant relationships.

